# Beyond the Glomerulus With Antineutrophil Cytoplasmic Antibody-Associated Vasculitis

**DOI:** 10.7759/cureus.75567

**Published:** 2024-12-11

**Authors:** Aiswarya Kosaraju, Sandhya Suresh, Ramprasad Elumalai, Barathi Gunabooshanam

**Affiliations:** 1 Nephrology, Sri Ramachandra Institute of Higher Education and Research, Chennai, IND; 2 Pathology, Sri Ramachandra Institute of Higher Education and Research, Chennai, IND

**Keywords:** anca-associated vasculitis, atypical p-anca, isolated tubulointerstitial anca vasculitis, peritubular capillaritis, renal vasculitis

## Abstract

Typical renal involvement of antineutrophilic cytoplasmic antibody (ANCA)-associated vasculitis (AAV) is pauci-immune glomerulonephritis that presents clinically as rapidly progressive renal failure (RPRF). Here, we report an unusual presentation of myeloperoxidase (MPO)-specific ANCA with isolated involvement of the tubulointerstitium in the form of peritubular capillaritis as the sole lesion without any involvement of the glomerulus. A 52-year-old woman with no previous comorbidities presented with nonspecific symptoms such as fatigue, dysuria, and nausea for two months. On evaluation, we found that she had renal failure (serum creatinine around 3-4 mg/dL) and numerous urinary pus cells with a sterile urine culture. We treated her with empirical antibiotics cover for culture-negative urinary tract infections. In view of nonresolving renal failure, we conducted a further evaluation and found that she had positive serum MPO titers, and renal biopsy revealed isolated tubulointerstitial involvement of the kidney with glomerular sparing in the form of acute interstitial nephritis (AIN). We observed peritubular capillaritis. We ruled out alternative explanations for AIN such as drug use and infections and attributed the peritubular capillaritis to the high titers of MPO positivity. We started her on steroids and mycophenolate mofetil (MMF) after ruling out infection. Over the next few months, she improved clinically and attained remission. Her creatinine reduced to within normal limits, and her MPO-ANCA titers became negative. She is currently on follow-up and doing well. The rarity of the entity should be considered when evaluating cases of AIN.

## Introduction

Antineutrophil cytoplasmic antibody (ANCA)-associated vasculitis (AAV) are a group of diseases that predominantly affect the small-sized arteries. The small blood vessels in any organ can be involved in microscopic polyangiitis (MPA) and granulomatosis with polyangiitis (GPA), but the kidneys and the upper and lower respiratory tracts are more commonly involved. They present with nonspecific symptoms such as fever, malaise, and anorexia. Specific organ involvement such as nasal crusting, otitis media, sinusitis, and nasal ulcers can be seen more commonly in GPA. AAV affects numerous organs and can cause cutaneous vasculitis, central nervous system involvement such as multiple mononeuropathy, cranial nerve abnormalities, ophthalmological involvement such as conjunctivitis, scleritis/episcleritis, retinal vasculitis, and renal involvement. Among these, renal involvement is more commonly seen in MPA than GPA and eosinophilic GPA (EGPA), which is also associated with higher morbidity and mortality rates [1]. In AAV, renal involvement often has the characteristics of rapidly progressive glomerulonephritis and presents as necrotizing glomerulonephritis, usually with resultant crescent formation. It rarely manifests as isolated tubulointerstitial nephritis (TIN) without apparent glomerular involvement [2]. The presence of ANCA as a biomarker aids in the preliminary diagnosis [3]. Anti-MPO and anti-proteinase 3 (PR3) antibodies can activate neutrophils in vitro, which is responsible for vasculitis [4,5]. TIN in ANCA is characterized by peritubular capillaritis (PTCitis). Treatment of isolated TIN in ANCA vasculitis remains unclear [6].

Here, we report a case of acute interstitial nephritis (AIN) as the sole renal lesion in myeloperoxidase (MPO) AAV without glomerulonephritis.

## Case presentation

This is the case report of a 52-year-old postmenopausal woman who presented to the outpatient room with chief complaints of progressive fatigue, nausea, dysuria, and decreased appetite in the last two months. The onset of fatigue was insidious and gradually progressive, inhibiting her from performing her routine activities. The onset of nausea was insidious and not associated with vomiting. The patient also complained of dysuria associated with frothy urine, yet we noted no gross hematuria. She had no history of fever, abdominal pain, decreased urine output, lower limb edema, or facial puffiness. She also did not have any symptoms such as skin rashes, joint pains, cough, shortness of breath, or hearing loss. On examination, her hemodynamics were stable and well within normal range with a normal systemic examination. There were no visible skin rashes. Her ear, nose, and throat examination was unremarkable.

Prior to her presenting at our institution, the patient was evaluated at a different hospital and diagnosed with deranged renal function parameters (serum creatinine: 3.49 mg/dL, urine routine showing plenty of pus cells, and protein of 2 to 3+ along with granular casts and were present) (Tables 1-2). In view of this, the patient underwent an ultrasound imaging of the abdomen and pelvis, suggestive of bulky kidneys. However, her urine culture was sterile. With a differential diagnosis of pyelonephritis, we treated the patient a culture-negative urinary tract infection with intravenous cefoperazone-sulbactum (renal-adjusted dose) for two weeks, following which the patient showed symptomatic improvement.

**Table 1:** Investigations gm/dL - gram per decilitre; mg/dL - milligram per decilitre; cmm - microlitre; meq/L - milliequivalent/litre; RBS - random blood sugar; ANA - antinuclear antibody; IF - immunofluorescence; anti-PR3 - anti proteinase 3

Investigation	Result	Reference range	Units
Hemoglobin	10.6	12-16	gm/dL
Total counts	9720	4,000-11,000	cells/cmm
Platelet count	3.43	1.5-4.5 lakhs	cells/cmm
RBS	120	70-140	mg/dL
Total bilirubin	1.0	0.1-1.2	mg/dL
Direct bilirubin	0.25	0-0.3	mg/dL
Serum albumin	4.2	3.2-4.8	g/dL
Blood urea nitrogen	43.2	7-18	mg/dL
Serum creatinine	3.4	0.6-1.3	mg/dL
Serum sodium	135	134-144	meq/L
Serum potassium	3.7	3.5-5	meq/L
Serum chloride	110	96-108	meq/L
Serum bicarbonate	25	21-29	meq/L
ANA by IF (1:180)	Negative	Negative	
C3	100	75-175	mg/dL
C4	30	15-45	mg/dL
ANCA by ELISA (Anti-MPO IgG)	Positive (33.96 U/mL)	>1 = POSITIVE	U/mL
ANCA by ELISA(Anti-PR 3 IgG)	Negative (12.26 U/mL)	>20 = POSITIVE	U/mL

**Table 2:** Urinalysis results HPF - high power field; mg/dL - milligram per decilitre; RBC - red blood cells

Urine analysis	Results	Reference range	Unit
Granular casts	Present	Nil	/HPF
Proteins	3+	Negative	<10 mg/dL
Glucose	2+	Negative	<10 mg/dL
Pus cells	Numerous	<5	cells/HPF
RBC	10	0-4	cells/HPF

After treatment, serum creatinine was reduced to 2.6 mg/dL, but this was short-lived. On examination, hemodynamics were stable and well within normal range with a normal systemic examination. Once the serum creatinine started showing an upward trend, the patient underwent urinalysis again, which revealed persistent granular casts, proteinuria, and sterile pyuria. Quantification of urinary protein was done with a urine protein creatinine ratio, and it was 1.8 mg/mg (sub-nephrotic range).

Serological workup, including C3 and C4 levels and ANA (by IF), indicated negative results. Using enzyme-linked immunosorbent assay (ELISA), we noted that MPO ANCA was positive: 33.56 U/mL (moderate to strong positive). To confirm the diagnosis, we performed an imaging-guided renal biopsy.

**Figure 1:**
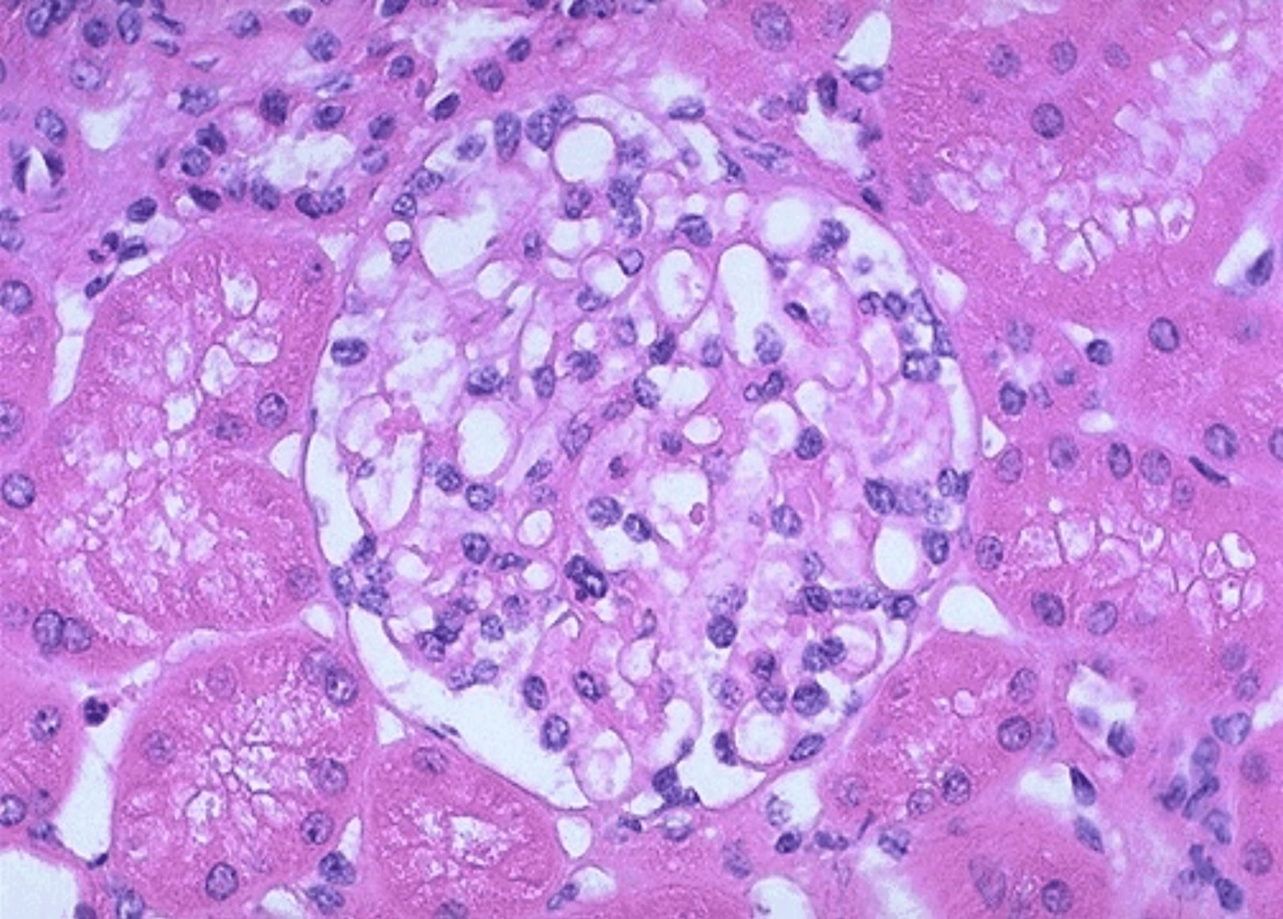
100x image H and E stain showing normal glomeruli surrounded by tubules showing vacuolation suggestive of tubular injury

**Figure 2:**
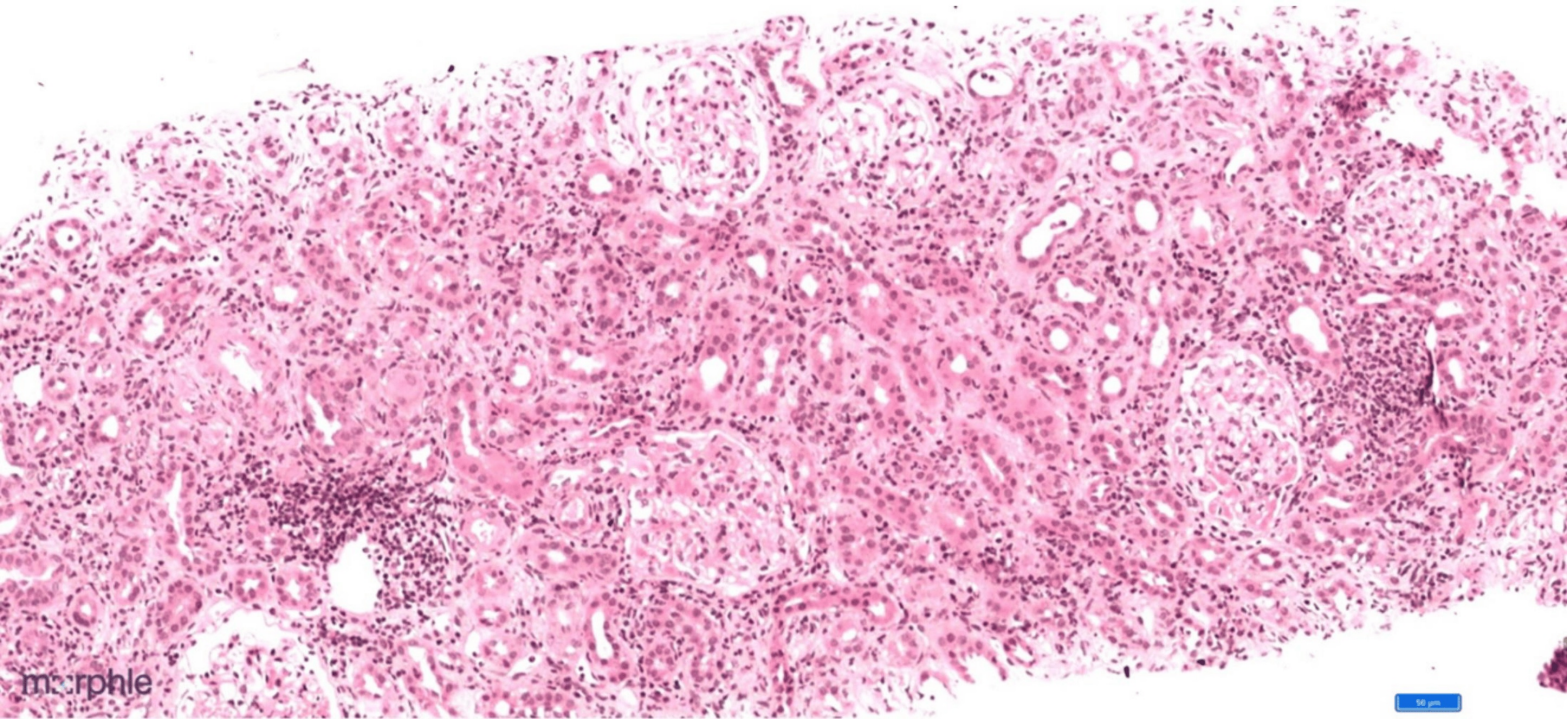
10x image hematoxylin and eosin stain showing interstitial inflammation

**Figure 3:**
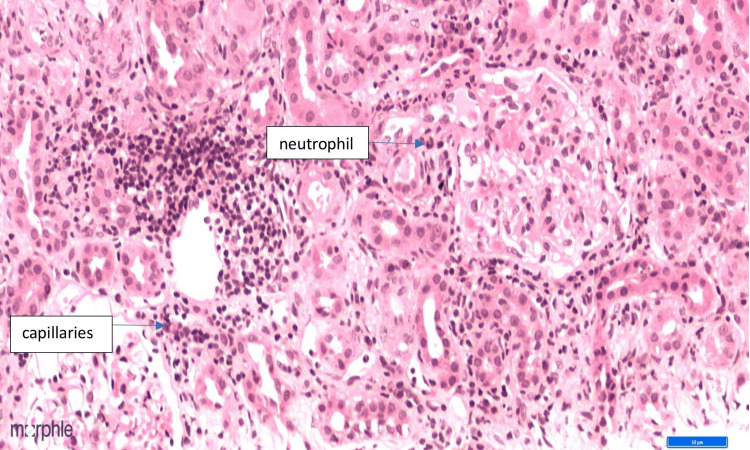
40x image of H and E stain showing peritubular capillaritis (as marked by the arrow) and interstitial inflammation with the presence of neutrophils (as marked by the arrow)

Renal biopsy histopathology revealed a surprising finding. Tubules revealed extensive injury with degenerative changes (nucleomegaly, prominent nucleoli, and mitosis). The renal cortical tissue sample had a total of 18 glomeruli, and glomerular sparing was noticed in all the 18 glomeruli, which is unusual when there is renal involvement in AAV (Figure 1). Additionally, we noted 10% of tubular atrophy. The interstitium showed diffuse inflammation (lymphocytes, plasma cells, neutrophils, and a few eosinophils) (Figure 2) with PTC (Figure 3). There were no red blood cell casts noted in the tubules.

We started the patient on treatment with oral steroids (1 mg/kg once a day) and MMF tablets (500 mg twice a day). We followed her up regularly in the outpatient services and monitored her serum ANCA levels using ELISA and serum creatinine. We tapered her steroids and stopped them over six months. We stopped her MMF tablets after one year of treatment when her ANCA titers remained undetectable for more than three months and her serum creatinine was 0.8 mg/dL. She continued to be on follow-up and remained in remission with stable renal function one year after stopping immunosuppression drugs.

## Discussion

Patients who have renal involvement in AAV are at significantly heightened risk of mortality during follow-up. Many previous studies concentrate on the severity of renal dysfunction in ANCA-AAV [7].

The present case was observed to be rare because the histopathology showed MPO AAV presenting with AIN as the sole renal lesion with no absolute glomerular involvement.

The typical renal lesion seen with AAV is acute necrotizing crescentic glomerulonephritis. While mononuclear tubulointerstitial infiltrates are also commonly reported in AAV, they are typically observed in association with glomerulonephritis [8]. There was no evidence of GN in our patient; she had isolated interstitial nephritis. The defining feature of tubulointerstitial damage in ANCA-AAV is PTC. According to Nakabayashi et al. [9], loss of CD34 vascular endothelial markers happens early in the course of the disease, indicating that activated neutrophils release MPO. This MPO, which releases proteolytic enzymes and radical oxygen species, causes tissue destruction in the peritubular capillary, namely, the lysis of endothelial cell membranes, as well as vascular basement membranes. This mechanism eventually further proceeds to the destruction of the peritubular capillary walls (vasculitis). This is thought to play an important role in the pathogenesis of TIN, which is associated with MPO-AAV [10]. Additional research is necessary to pinpoint the precise pathophysiology of AAV-associated interstitial nephritis.

Among AAV patients, 54.5% have PTCitis or arteriolitis, independent of whether they have crescentic glomerulonephritis, according to Ohashi et al. [10]. Additionally, there is a strong correlation between the existence of PTCitis and the level of urine α1-microglobulin secretion, indicating that PTCitis is the primary cause of tubular damage in AAV [10]. An intriguing case study on second renal biopsies demonstrates that, during the AAV patient’s clinical course, PTCitis occurred before glomerulonephritis.

Compared with AAV-associated typical glomerulonephritis, isolated interstitial nephritis AAV cases presented with much less severity and demonstrated significantly fewer urinary red blood cells, lesser 24-hour urine protein, and lower levels of serum creatinine and serum MPO titers.

Clinical management of AAV-associated interstitial nephritis is complicated by the fact that it is a rare presentation of AAV that lacks well-recognized diagnostic standards and treatment protocols. The therapy for AAV mainly involves glucocorticoids, immunosuppressants, and rituximab. In cases of isolated TIN, there are no standard guidelines. According to older literature, physicians have treated it with a relatively lesser dose of immunosuppressant and noticed that patients responded well. They noticed that isolated TIN AAV patients had attained remission and remained in it much better compared to those of necrotizing crescenteric AAV.

Plafkin et al. [11] established that the renal biopsy indicated acute interstitial nephritis characterized by significant fibrosis, tubular atrophy, and glomerulosclerosis, without evidence of active glomerular illness. MPO titers persisted at 132 U/mL, prompting the initiation of mycophenolic acid and prednisone without effect, subsequently leading to the administration of rituximab for AAV-associated AIN. Serum creatinine increased to 6.0 mg/dL, and the patient commenced peritoneal dialysis. They advised that other reasons for AIN, such as proton pump inhibitor usage, should be cautiously evaluated in the context of high-titer ANCA positive.

A few AAV patients with acute interstitial nephritis did progress to crescentic glomerulonephritis (cardiorenal syndrome type 1 (CRS1)) [12]. Treatment with cimetidine and other unknown mechanisms were their confounding factors. Thus, all patients should be on close follow-up with regular monitoring of renal function and MPO-ANCA titers.

In our patient, we also evaluated all other possible causes of interstitial nephritis. We stopped drugs including antibiotics, and we ruled out infection. The patient underwent a biopsy because the creatinine remained elevated despite these measures. We attributed interstitial nephritis with classical PTCitis in the setting of MPO-ANCA positivity to ANCA AAV. The possibility that the glomerular involvement was focal and was not sampled by the biopsy was also considered, but the sample had 18 glomeruli, and all the 18 glomeruli were unaffected, hence making it implausible.

## Conclusions

AAV may manifest as solitary AIN without glomerular involvement. The infrequency of this presentation may hinder timely treatment. A high index of suspicion is necessary to diagnose it early and initiate immunomodulators. All patients with biopsy showing interstitial nephritis and PTCitis should also be evaluated for ANCA positivity. Observing and monitoring the response can help in deciding the response to initiated treatment and the need for additional drugs.
